# Experimental and Clinical Applications of Red and Near-Infrared Photobiomodulation on Endothelial Dysfunction: A Review

**DOI:** 10.3390/biomedicines9030274

**Published:** 2021-03-09

**Authors:** Esteban Colombo, Antonio Signore, Stefano Aicardi, Angelina Zekiy, Anatoliy Utyuzh, Stefano Benedicenti, Andrea Amaroli

**Affiliations:** 1 Laser Therapy Centre, Department of Surgical and Diagnostic Sciences, University of Genoa, 16132 Genoa, Italy; esteban.colombo92@gmail.com (E.C.); dr.signore@icloud.com (A.S.); stefano.benedicenti@unige.it (S.B.); 2 Department of Therapeutic Dentistry, Faculty of Dentistry, First Moscow State Medical University (Sechenov University), 119991 Moscow, Russia; 3 Department for the Earth, Environment and Life Sciences, University of Genoa, 16132 Genoa, Italy; stefano.aicardi94@libero.it; 4 Department of Orthopaedic Dentistry, Faculty of Dentistry, First Moscow State Medical University (Sechenov University), 119991 Moscow, Russia; zekiy82@bk.ru (A.Z.); anatoliy.utyuzh@gmail.com (A.U.)

**Keywords:** low-level laser therapy, phototherapy, endothelium, vascular disease, healing, angiogenesis, ischemia, hypertension, inflammation, nitric oxide

## Abstract

Background: Under physiological conditions, endothelial cells are the main regulator of arterial tone homeostasis and vascular growth, sensing and transducing signals between tissue and blood. Disease risk factors can lead to their unbalanced homeostasis, known as endothelial dysfunction. Red and near-infrared light can interact with animal cells and modulate their metabolism upon interaction with mitochondria’s cytochromes, which leads to increased oxygen consumption, ATP production and ROS, as well as to regulate NO release and intracellular Ca^2+^ concentration. This medical subject is known as photobiomodulation (PBM). We present a review of the literature on the in vitro and in vivo effects of PBM on endothelial dysfunction. Methods: A search strategy was developed consistent with the PRISMA statement. The PubMed, Scopus, Cochrane, and Scholar electronic databases were consulted to search for in vitro and in vivo studies. Results: Fifty out of >12,000 articles were selected. Conclusions: The PBM can modulate endothelial dysfunction, improving inflammation, angiogenesis, and vasodilatation. Among the studies, 808 nm and 18 J (0.2 W, 2.05 cm^2^) intracoronary irradiation can prevent restenosis as well as 645 nm and 20 J (0.25 W, 2 cm^2^) can stimulate angiogenesis. PBM can also support hypertension cure. However, more extensive randomised controlled trials are necessary.

## 1. Introduction

An increase in the prosperity and the prevention of childhood mortality through socioeconomic and scientific progress has led to a shift, over the last two decades years, from risks for infancy diseases towards those for adulthood diseases [1,2].

Indeed, the main disease risk factors implicated for worldwide deaths are hypertension (approximately 7–18%), ischemic heart disease (approximately 5–14%), and cerebrovascular disease (approximately 4–14%), which are all threats of cardiovascular illness [3].

The endothelium is a monolayer of endothelial cells cladding the lumen of the vascular beds of the entire cardiovascular system, from the heart to the smallest capillaries.

It is a metabolically and mechanically dynamic organ, separating the vessel wall from the blood- and its components [4]. The endothelium, which was for a long time considered to be a relatively inert cellular monolayer, has recently been recognised as an important modulator of key physiological functions [1].

The endothelium’s role is carried out through membrane-bound receptors for hormones, proteins, metabolites, lipid transporting particles, as well as through specific junctional proteins and receptors that regulate cell–cell and cell-matrix interactions [5,6].

Recently, the important regulatory role of mitochondria on endothelial cells’ cytoprotective phenomena have been evidenced. In addition, stimuli affecting mitochondrial dynamics of endothelial cells can affect the production of reactive oxygen species (ROS) and adenosine triphosphate (ATP), leading to physiological or pathological response [7]. Additionally, endothelial functions may also depend on changes in intracellular calcium (Ca^2+^) concentration, depending on depletion of Ca^2+^ stores and in-out-in Ca^2+^ signalling events, as well as multiple transient receptor potential (TRP) channel isoforms activation [8,9].

Under physiological conditions, endothelial cells are the main regulators of arterial tone homeostasis and vascular growth, sensing and transducing signals between tissue and blood [6]. Endothelial cells, through different antiplatelet and anticoagulant mechanisms, counteract thrombosis and, by regulating the expression of binding sites for procoagulant and anticoagulant agents, control the clotting system. Additionally, platelet adhesion to leukocytes represents the initial stage leading to exudation of leukocytes to inflammation or infection areas, followed by a platelet–leukocyte interaction and aggregation, and then vascular occlusion [6]. Lastly, endothelial cells, thanks to several vasoactive substances such as endothelin, angiotensin, nitric oxide (NO), vascular endothelial growth factor (VEGF), for instance, exert a regulatory role in vessel tone and growth, as well as cell proliferation and angiogenesis [1,5,6].

The dynamic features of the endothelium permit rapid response to diverse stimuli such as coagulation proteins, microbial components, shear stress, growth factors, and cytokines. These “activation” responses have evolved for host defence against microorganisms and the repair of tissue injury and are generally localised and beneficial [10].

However, this well-balanced endothelial regulation of vascular function can be affected by several disease risk factors, leading to both unbalanced homeostasis and an unphysiological state of healthy endothelial cells, which is known as endothelial dysfunction. As a consequence, endothelium shows reduced vasodilation induction, experiences a proinflammatory state, and prothrombic properties, which are associated with most forms of cardiovascular diseases, such as coronary artery disease, hypertension, peripheral vascular disease, chronic heart failure, chronic kidney, diabetes, failure, and severe viral infections [10].

However, as carefully reviewed by Daiber et al. [1], the evidence that the progression of endothelial dysfunction can be counteracted and reversed increases the possibility of retarding and, in some cases, preventing the progression of the related diseases and encourages efforts to explore new therapies.

Routinely, treatments applied for patient care have focused on indirect pleiotropic antioxidant properties and modulating NADPH oxidase enzymes and mitochondrial activities to prevent endothelial nitric oxide synthase (eNOS) uncoupling and stimulating anti-inflammatory effects, leading to improved endothelial function. Pharmacological agents can also be employed to equilibrate the oxidatively impaired activity of soluble guanylyl cyclase of smooth muscle [1].

In addition, mitochondria- and calcium-targeted therapeutics to counteract oxidative stress and improve endothelial dysfunction in cardiovascular disease have also been developed [11].

As fully detailed in the next paragraph, light in the red and near-infrared range of wavelength can interact with animal cells and can modulate their metabolism. Indeed, studies conducted by our research team have shown that modulation in oxygen consumption, ATP production, and ROS, after interaction of those wavelengths with mitochondrial cytochromes, as well as light, can regulate the NO release and the intracellular Ca^2+^ concentration [12,13,14,15]. This medical topic has been defined as low-level laser therapy, but recently, the more appropriate definition of photobiomodulation (PBM) was introduced [14].

Photobiomodulation can, thereby, counteract inflammation, stimulate growth factor expression, and modulate many cellular pathways [12,14] by different strategies, as described in the paragraph below. Therefore, PBM can support clinicians and their patients to manage and experience faster healing. In this review, we explore the ability of red and near-infrared (NIR) light to modulate cell homeostasis with particular attention to the endothelial cell line, in vitro. Additionally, the in vivo therapeutic effects of PBM on animal and human subjects are discussed. In particular, we present a review from the cell to patient literature on the effect of PBM on endothelial dysfunction.

### 1.1. Molecular Targets of Red and Near-Infrared Light: Primary and Secondary Effects

Interactions of light at the visible-red and near-infrared wavelengths with non-plant cells have been described [12]. Animal cells interacting with light evolved the ability to use specific light stimuli for vitamin-D production and vision, as examples. Although animal cells did not choose sunlight as a source of energy for their metabolism, a process close to photosynthesis, i.e., light–cell interaction can also take place in cells. This process is referred to as photobiomodulation and describes the ability of photons to interact with atoms and molecules within the cell and induces biological modulation on tissue homeostasis [12]. Specifically, following the standard model of particle physics, i.e., a paradigm of quantum field theory, the four known forces are settled by a fundamental carrier particle, the boson. Photons, belonging to the family of bosons, are the core particle of light and they can carry electromagnetic force [16]. Therefore, they can behave as a power source, where the brightness of light describes the photons’ number and their colour is the result of energy contained in each photon. In this way, photons are theoretically able to interact with cells, and then energetically modulate the homeostasis of the tissue [12,16]. However, to make photobiomodulation happen, an energy conversion needs to take place. Indeed, molecules in living systems can absorb photons’ energy, reaching an electronically excited state that temporarily modifies their conformation and function.

The uptake of photons’ energy by cells needs biomolecules that may undergo an excited state [12]. In the life forms, two types of molecules exist, those specialised to absorb light, such as the photoreceptors, and non-specialised molecules, such as the photoacceptors. The latter are more common than photoreceptors and are part of ubiquitous metabolic pathways not directly related to light processing [17]; this explains how photobiomodulation can affect key cellular pathways of all life forms, from protozoa to humans [12].

Not all molecules feature light-acceptor ability. Organic cofactors or metal ions are fundamental elements that allow excitation of molecules from the ground state to an excited state and their change in conformation and function [18].

Water deserves a separate discussion, as a polar molecule with more hydrogens than atoms, as well as nonlinear and time-dependent chaotic behaviours, because it display extremely complex vibrations. As effectively discussed by Santana-Blank and collaborators, water was considered to be an innocuous medium for a long time [16]. However, recently its key role in physiologic mechanisms has been reconsidered, and therefore the possibility that external radiant energies may route its organisation and selectively lead biological function. For this reason, water in the 600–1100 nm range, can behave as a biological photoacceptor, despite its relatively low coefficient of absorption, and therefore can lead and improve biological reactions [16,19].

**Figure 1:**
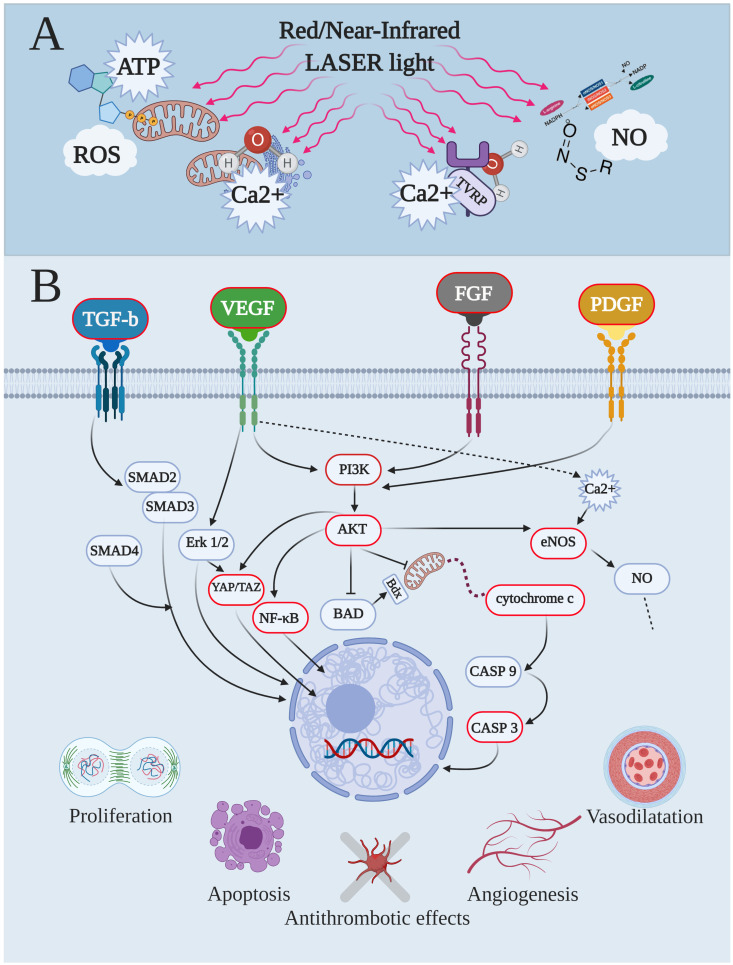
Endothelial-cell pathways modulated by red and near-Infrared laser lights, primary targets and secondary effects. As the first step, both red and near-infrared light interacted with the primary target, inducing immediate photochemical changes in the photoacceptor. This controlled the stimulation of the primary effects (**A**), such as the modulation’s levels of adenosine triphosphate (ATP), reactive oxygen species (ROS), intracellular calcium concentration (Ca^2+^), and nitric oxide (NO). The primary effects lead to the secondary effects (**B**) of photobiomodulation, which involve expression or activation of second messengers, modulation of gene expression and enzyme activities, and then signalling pathways, which resulted in physiological events such as proliferation/healing, apoptosis, antithrombotic effects, angiogenesis, and vasodilatation (**B**), as examples. AKT, alpha serine/threonine kinases; BAD, BCL2 associated agonist of cell death; CASP, caspase; eNOS, endothelial-nitric oxide synthase; Erk, extracellular signal-regulated kinase; FGF, fibroblast growth factor; YAP/TAZ, yes-associated protein/transcriptional coactivator with PDZ-binding motif; Nf-κB, nuclear factor-κB; PI3K, phosphoinositide 3-kinases; PDGF, platelet-derived growth factor; SMAD, small mother against decapentaplegic; TGF-b, transforming growth factor-b; VEGF, vascular endothelial growth factor.

Among the different molecules within cells, haemoglobin is a well-known photoacceptor, which reacts with visible and NIR light based on wavelengths and its redox state. Additionally, behaviour such as photoacceptors was also described in other cellular molecules such as catalase, cytochromes, cryptochromes, nitric oxide synthase, nitrosothiols and di-nitrosyl iron complexes, and superoxide dismutase [17,18]. Therefore, since many of these photoacceptors were described in the mitochondria, it is not strange that this organelle is considered to be the elective cellular target to explain the light and animal–cell interaction for photobiomodulation. The parallel and convergent evolution of both chloroplasts and mitochondria, from ancestral bacteria, has to be also taken into account to explain mitochondria’s ability for responding to light stimuli [12]. Experiments have demonstrated that isolated mitochondria are sensitive to red and NIR spectrum and that the interaction increases ATP synthesis and oxygen consumption, as well as ROS generation, Ca^2+^ modulation, and photodissociation of NO from cytochrome c oxidase [12]. In particular, the cytochrome c oxidase (complex IV) has been shown to be activated in vitro by a red laser (633 nm) [20]. According to metal-ligand systems and absorption spectra, such as 450, 620–680, and 760–895 nm, characteristically different peaks may be related to it [21]. In previous papers, we have shown that 808 nm stimulated complex IV electively and that complex III was excited poorly, while complexes I and II were not affected [12,22]. At 980-nm the interaction behaved as a window effect and it interested complexes III and IV [23]. In addition, by increasing the wavelength to 1064 nm, complexes I, III, and IV were influenced, while the extrinsic mitochondrial membrane complex II seems again to be not receptive to photons at this wavelength [24]. Lastly, red and NIR light exposure induces NO release from S-nitrosothiols and di-nitrosyl iron complexes in a wavelength-dependent modality [25], as well as NO concentration, which can be changed by modulation of eNOS activity and expression after light exposure [13,26]. Lastly, Wang et al. [27] showed, for the parameters tested, that 980 nm affected temperature-gated Ca^2+^ ion channels, also probably via water resonance influence and transient “heating”. Altogether, this direct and immediate photochemical change in the photoacceptor’s primary targets by light can be considered to be primary effects [12,17] (Figure 1A). They lead to the secondary effects of photobiomodulation, which involve the expression or activation of second messengers, modulation of gene expression and enzyme activities, and then signalling pathways that result in macroscopical physiological consequences [12,17] (Figure 1B). In this way, the PBM, irradiated by intravenous, transmucosal, transcranial, or transcutaneous mode, can affect, after minutes as well as days, the homeostasis of tissue and represents a novel paradigm to treat altered physiological conditions [12,14,17].

## 2. Materials and Methods

Our review was carried out in compliance with the PRISMA guidelines (Supplemental Material Figure S1). Papers were independently searched by four authors (A.A., E.C., A.S., and S.A.) on the PubMed, Scopus, Cochrane, and Scholar databases. The following keywords were applied to meet the strategy investigation: “low-level laser therapy” OR “photobiomodulation” OR “laser phototherapy” AND “endothelial OR “endothelial dysfunction” OR “endothelium” OR “vascular” OR “vascular dysfunction”. Additional studies were also identified from the references. Articles were listed and duplicates were deleted by all the authors as a consequence of the large number of papers identified. We also initially screened the works by title and abstracts according to inclusion and exclusion criteria. The inclusion criteria included the following: (1) studies published in English in journals with a peer-review process before publication; (2) works published before 1 September 2020; (3) studies that complied with the topic of the review; (4) a clear description of the type of light emitting diode (LED) or laser device and treatment parameters employed; (5) therapies were immediately traceable to PBM; (6) type of articles such as original research, case reports, and short communications; and (7) articles drafted according to “parameter reproducibility in photobiomodulation” by Tunér and Jenkins [28]. The exclusion criteria included the following: (1) in vitro studies on stem cell or cell line not referring to endothelium; (2) LED or laser therapies not adhering to the principles of PBM; (3) studies not focused on the topic of the review; and (4) types of articles such as reviews, abstracts to congress, and patents. The selection process is available in Supplemental Material Figure S1.

## 3. Results

Fifty out of >12,000 articles selected by PubMed, Scopus, Cochrane, and Scholar, as well as from article references were judged to be eligible for the review (Supplemental Material Figure S1). Actually, research by Scholar was the most dispersive, and no paper after the first 150 papers screened was included in the review. Conversely, PubMed resulted in being the most inclusive and Scopus and Cochrane showed similar useful support.

Following a previous review of PBM on bone socket preservation [14], the most frequent reason for exclusion was the unsuitability with the parameter reproducibility in photobiomodulation described by Tunér and Jenkins (2016) [28]. Four papers out of 54 were rejected from the eligibility because they did not completely fit the inclusion criteria after the full texts were read.

Unfortunately, only 17% of the studies had used a power meter to measure the power at the target, and in many studies, the probe used for irradiation was not clearly described, as well as only a few of the studies clearly described the PBM’s parameters in the abstract.

### 3.1. In Vitro Studies

Concerning the in vitro studies (Table 1), ~50% of the experiments were performed on human umbilical vein endothelial cell lines (HUVEC, 62.5% were umbilical vein primary cell culture and 37.5% were immortalised cells), while 29% of the studies used other primary cell cultures such as human endothelial cells isolated from coronary vessels (HuC EC), neonatal rat ventricular myocytes (RVM-neo), rabbit aorta endothelial cell line (RAEC), human adjacent annulus fibrosus cells (h-AFC), and human pulp fibroblasts HPF. Lastly, ~23% of the experiments were performed on other transformed cell lines such as mouse NCTC clone 929 Clone of strain L (L929), permanent human umbilical vein cell line (EA.hy926), human dermal microvascular endothelial cells (HMVEC-d), and human endothelial cells from the umbilical cord (HECV).

Except for two studies, the experiments were performed in continuous wave mode of irradiation, and an LED device was used in only one study instead of a diode laser. Lastly, the irradiation was prevalently performed by the visible red light; no study showed an adverse effect.

As described in the previous Section 1.1, PBM is the result of an interaction between light and photoacceptors (primary target, Figure 1A), which is followed by a succession of the primary (Figure 1A) and secondary effects (Figure 1B), and physiological events. Therefore, the results at the cellular level, summarized in Table 1, are considered to be secondary effects (Figure 1B) in response to molecules such as ATP, ROS, Ca^2+^, and NO (Figure 1A).

The PBM affects the VEGF family and their receptors [29,31,32,35,36,41], as well as anti-inflammatory effects are evidenced by the impact on matrix metalloproteinases (MMP), tumour necrosis factor (TNF-α), interleukin (IL)-6, and IL-8 [30,37]. Additionally, a modulation on NO and ATP production was shown [26,33,43,44] and inhibitory and stimulatory effects on apoptosis [26,42] and proliferation/viability of the cells [30,34,38,39,40,44] were suggested, respectively.

### 3.2. In Vivo Preclinical (Animal) Studies

Except for one study on rabbits and one on hamsters, the preclinical experiments were carried out on murine models (Table 2). 

Sixty-six per cent of the studies investigated wound healing and angiogenesis, 21% of the studies were on ischemia, and only 13% of the studies were on blood pressure and micro- and macrocirculation. More than 70% of the studies used visible red light and in 87% of the studies, the light was irradiated with a diode laser. The screening on the literature of preclinical studies confirmed that the evidence shown was from in vitro experiments. No side effect was experienced by animals because of PBM, while angiogenesis was experienced. The data can be catalogued in three macro studies, such as wound healing and angiogenesis, ischemia, and blood features (both circulation and pressure). Basically, modulation of VEGF family proteins [46,47,50,51,52,53,55,57,61,65], anti-inflammation [46,49,51,53,54,55], and nitric oxide production [45,47,49,56,66,67] were described.

### 3.3. Clinical Studies

Taken together, the clinical studies showed interesting effectiveness of PBM on endothelial dysfunction, not accompanied by adverse effects.

One hundred per cent of humans exposed to PBM showed positive effects (Table 3). Among the nine studies selected, one study was a randomised controlled trial, one study was a randomised trial, and the remaining were pilot studies. In three studies [69,70,71], patients experienced prevention of prodromal complications in saphenectomy post myocardial revascularization and improvement of recovery after coronary intervention by modulation of endothelin, NO derivates, and transforming-growth-factor-b. In three studies [72,73,74], inflammation was reduced, as a consequence of antioxidants increment and tumour necrosis factor decrement, as well as in two studies the ischemia damages were counteracted by new microvascular restoration and increased cognitive performances. In one study blood-pressure decreased, and thus hypertension was ameliorated. Lastly, the studies pointed out the angiogenesis effect of PBM through the modulation of the VEGF-family, in accordance with the in vitro and preclinical data. In contrast to the previous in vitro and preclinical studies, five out of nine studies on humans were performed with NIR light, the remaining four studies were conducted with red-light and no study used an LED device.

## 4. Discussion

### 4.1. In Vitro Studies

Concerning endothelial cells, and more generally, the eukaryotic cell, ATP, ROS, Ca^2+^, and NO molecules are responsible for their metabolic behaviour [12]. Substantially, the modulation of Ca^2+^ homeostasis tends to regulate growth factor release, such as fibroblast growth factors (FGF), transforming growth factor (TGF)-b, VEGF, and platelet-derived growth factor (PDGF) [77,78], as well as ATP, ROS, and NO can act as proangiogenic factors or inducers of cell alteration, according to their concentrations and target [79,80,81]. The VEGF family and their receptors are influenced by PBM, as evidenced by ELISA, qRT-PCR, and immunoblot investigations on different cell lines [29,31,32,36,41]. In particular, 632 nm and 0.63, 1.05, 2.1, or 4.2 J (0.0035 W, 2 cm^2^) increased VEGF secretion, 8.4 and 12.6 J decreased VEGF secretion, while 0.21 and 6.3 J did not have an effect [29]. Additionally, 660 nm and 0.10 J (0.01 W, 0.04 cm^2^) and 0.15 J (0.015 W, 0.04 cm^2^) increased secretion of VEGF-A, VEGF-C, VEGF-D, and VEGF-receptor (R)1 [32], as well as reduction of VEGF-A, sVEGFR-1, and sVEGFR-2 by irradiation with 635 nm and 160, 320 or 640 J (0.15 W, 80 cm^2^) was observed by Góralczyk et al. [36]; 904 nm and 200 or 300 J (50 W, 0.01 cm^2^), in a pulsed mode of irradiation, incremented VEGF expression [41]. The VEGF family constitutes five different types of protein signalling molecules, i.e., VEGF-A or VEGF, VEGF-B, VEGF-C, VEGF-D, and placental growth factor (PlGF), which plays a key role in endothelial tissue homeostasis. The link of these molecules with their specific receptors (VEGFR-1, -2, -3) leads to the development of both the cardiovascular system and the angiogenesis, with a beneficial effect on peripheral arterial disease, ischemic heart disease, healing, coagulation, and also the female reproductive cycle [82]. Unfortunately, VEGF is expressed in cancers and VEGF inhibition is considered to be a promising treatment option, stopping tumour metastasis and growth [83]. Therefore, the chance of modulating the VEGF expression by adequate laser therapy parameters could support the clinician for different stimulatory or inhibitory curative approaches on endothelial dysfunction, according to need.

The analysis of the selected literature suggests that the support by PBM to endothelial dysfunction can additionally be traced to an anti-inflammation effect, which leads to an increase in cell viability, similar to the recent description of pre-osteoblast cell line by Hanna et al. [84]. Hwang et al. [31] showed that 645 nm and 32 or 64 J (0.010 or 0.012 W, 2.8 cm^2^) inhibited inflammatory mediators and catabolic enzymes, such as matrix metalloproteinase (MMP)-1, MMP-3, and brain-derived neurotrophic factor (BDNF), and MMP-1, MMP-3, BDNF, and interleukin (IL)-8, respectively. Góralczyk et al., [37] described a reduction of inflammation by a decrease in TNF-α and IL-6 after irradiation with 630 nm/160 J (0.15 W, 80 cm^2^) or 830 nm/160 J (0.3 W, 80 cm^2^).

Nitric oxide and ATP are ambiguous molecules acting as proinflammatory or anti-inflammatory mediators according to their concentration, target, and time of exposure [85,86]. Therefore, they are able to downregulate or upregulate some MMPs synthesis and affect TNF and proinflammatory markers [85,86,87]. In this context, the ability of laser light to induce NO production or release and to change the energetic cell metabolism, such as the ATP production [25,26,44], can control the proinflammatory molecules. This behaviour may lead to a reduction of apoptosis’ indices such as caspase 3, annexin, and cytochrome c [26], as well as protects against TNF/cycloheximide (CHX)-induced apoptosis pathway by inhibiting p38 mitogen-activated protein kinase (MAPK) and nuclear factor kappa-light-chain-enhancer of activated B (NF-κB) signals [42]; also, according to De Nadai et al. [88]. Undoubtedly, the strengths of the in vitro studies selected for our review are the prevalent use of human umbilical vein endothelial cells (HUVEC) cell line and a wide panel of markers also investigated on primary cells. Therefore, despite the fact that different light therapies were administered in the experimental setup, we can try to reveal a pathway of the effectiveness of red and NIR light. The primary interaction between red and NIR light and the photoacceptors into the cell (see [12]) leads to a modulation of the VEGF molecules family, including PDGF, and the FGF and their receptors. The following step appears to involve the phospho-inositide 3-kinase (PI3K) protein, which can interact with the protein kinase B (AKT), a multiple-process cellular protein, able to have a key role in eNOS synthesis [33], NF-kB signal, and inhibition of mitochondrial proapoptotic triggering, via the cytochrome c and caspase 3 pathway. In this way, the cell’s fate can be routed through cell proliferation and angiogenesis, or apoptosis, but also an antithrombotic effect can be stimulated (see Table 1). Likewise, the TGF-b signal involving small mother against decapentaplegics (SMADs), a family of structurally similar proteins, may be involved, as well as AKT can take the transcriptional regulator integrating mechanical signal yes-associated protein (YAP)/tafazzin (TAZ) [35], which modulates aspects of cell behaviour, including cell plasticity, proliferation, and stem cell differentiation, essential for tissue regeneration [89]. Additionally, the NO release mediated by red/NIR light through an effect on eNOS expression, the calcium–calmodulin interaction, or the gas dissociation from S-nitrosylated proteins can be involved in an intracellular NO dose-dependent mechanism, as also shown in ex vivo experiments [90]. Lastly, wavelength comparison studies indicate 635 nm as the best effector of endothelial cell proliferation with respect to 808 nm in standard growth condition, while in a like-diabetic hyperglycaemic environment, 808 nm seems to be more effective, probably because of the involvement of different pathways via either NO or mitochondria’s metabolism, as suggested by authors [25,42,44]. It is knownthat a cellular redox state has a role in the sensitivity of cells to PBM and can, therefore, explain the different responses to phototherapy by diabetic cells; diabetic pro-oxidant cells are generally differently more sensitive to PBM thanks to oxidation of cytochrome c oxidase and the increased mitochondrial responsiveness [91].

### 4.2. In Vivo Preclinical (Animal) Studies

In rat model PBM, different wavelengths and laser parameters can improve the healing process in injured muscle and skin; the PBM affects the inflammation process by modulation of its markers.

Basically, 660 nm visible light and 2 J (0.04 W, 0.04 cm^2^) or (0.03 W, 0.028 cm^2^) decreased cyclooxygenase (Cox)-2 [52] and IL-6, and MMP-3 and -9 [54], respectively.

Infrared light, 904 nm, irradiated in pulsed mode with 3 J on 0.1 cm^2^ (output power = 0.04 W) or 0.4 J on 1.77 cm^2^ (output power = 0.00078 W), prevented IL-6 [53] formation and has decremented ROS, NO, lipid peroxidation, protein carbonylation, and other proinflammatory markers [56]. Conversely, anti-inflammation indicators such as IL-10 [53] and superoxide dismutase, catalase, and glutathione peroxidase (GPx) were improved [56].

As previously seen through in vitro experiences, different signal pathways influence VEGF family, which is modulated. The same behaviour is observed in the angiogenesis experimental purposes, in which, as described in Table 2, PBM modulated both cytokines and metalloproteinases [51,53,54,55,61,65], and the collagens and blood vessels took form [54,55,62,65]. The only comparative work pointed out that light coherence was not shown to be essential to angiogenesis [59]. Nevertheless, in only one study, the data contextualised with the conclusion of Hode [92] by which coherence seemed to not be a potentially important factor in the overall efficacy of photobiomodulation, particularly in a clinical setting.

Additionally, the increment of NO production or release observed in cell culture also took place in rats’ model and led to a reduction of systolic arterial pressure, induced a long-lasting hypotensive effect, and improved heart rate [66,67]; 660 nm and 5.6 J (0.1 W, 0.058 or 0.028 cm^2^) irradiated transcutaneously in six points, seems to represent an effective therapy.

Lastly, modulation of NO and improvement of angiogenesis could also diminish myocardium inflammation, as well as enhanced ischemic revascularisation and angiogenesis, which increased tissue viability and reduced infarct size [46,47,49].

The information experienced by in vitro and preclinical experiments showed coherent results and suggested a promising clinical opportunity for therapeutic approaches on humans.

### 4.3. Clinical Studies

Thanks to both a randomised controlled trial (RCT) [69] and a randomised trial (RT) study [70] with a six-month follow-up, Derkacz’s team strongly demonstrated that 808 nm and 18 J (0.2 W, 2.05 cm^2^) intracoronary irradiation, during percutaneous coronary interventions, can counteract restenosis cascade. It has been assumed that growth factors play a role in the restenosis process stimulation. Mitogenic smooth muscle cell expression by VEGF and FGF-2 support, in particular, migration and constitution of neointima [93]. The Darkacz et al. therapy [69,70] stimulated NO release and decreased FGF-2 concentration as well as the expression, in the more advanced stage, of endothelin-1, a protein associated with the process of coronary restenosis after percutaneous transluminal coronary angioplasty [94]; the effect led to smaller neointima formation and prevented the process of restenosis. Despite a different approach and a lower number of patients but with the same six-month follow-up, an improved effect of PBM was also shown by Pinto et al. [71] on saphenectomy post-myocardial revascularisation irradiated with 780 nm/0.75 J (0.025 W, 0.04 cm^2^).

Concerning healing and angiogenesis, clinical data was in agreement with the in vitro and on preclinical positive evidence, but the different purposes and therapy parameters impeded a successful comparison among the works.

However, Angiero and co-workers [73] showed interesting results on a high number of patients, and a 21-day follow-up, thanks to irradiation on two points at baseline and after 1, 3, and 7 days with 645 nm and 20 J (0.25 W, 2 cm^2^). Patients experienced a reduction of bradykinin (a vasoactive peptide involved in the classical signs of inflammation), local heat, redness, pain, and swelling [95] as well as VEGF, while EGF (a growth factor implicated in wound healing [96]), increased; they concluded that patients with periodontitis had a beneficial effect due to PBM, in the early phases of the revascularisation and healing.

Maksimovich et al. [75] and Vargas et al. [97], through two different wavelengths in the visible (633 nm) and infrared (1064 nm) spectrum, showed in humans the improvement effect of PBM on ischemia, as previously described on rat models. Indeed, PBM on patients affected by cerebral ischemia restored cerebral collateral and capillary blood supply, improved microcirculation, recovered cellular and tissue metabolism, stimulated neurogenesis, and caused regenerative processes, if irradiated by different energy from 29 to 106 J and 633 nm [75]; no side effects were observed in the irradiated patients with follow-up for two-years. In addition, patients with altered neurocognitive function, because of ischemia, incremented cognitive performance, improved carotid artery intima-media thickness, and increased resting-state EEG alpha, beta, and gamma power, by an increment of prefrontal blood oxygen level after irradiation with 1064 nm and 816 J (3.4 W, 13.6 cm^2^) [97].

Lastly, although only a paper was selected after inclusion and exclusion criteria as well as research by keywords, PBM could provide support for patients with hypertension. Endothelial dysfunction, which is characterised by an impairment of nitric oxide (NO) bioavailability, is indeed an important risk factor for both hypertension and cardiovascular disease and may represent a major link between such conditions. Mitchell and Mack [98] demonstrated near-infrared light in the form of low-level laser therapy increased NO levels in venous blood draining from the treatment site in healthy subjects. As previously discussed, the preclinical studies on animal model showed effective therapeutic PBM’s windows to reduce pressure and heart rate and induce a long-lasting hypotensive effect. Additionally, intravascular laser irradiation of blood discovered by Russian scientists in the 1970s, as well as plasmapheresis of blood irradiated with PBM, showed improvement in patients with hypertension disease by normalisation of plasma levels of stable nitric oxide [99,100]. This was contextualised by the work of Kovalenko et al. [76], where patients affected by hyperuricemia and high blood pressure showed beneficial effects using PBM with 1.35 J, (0.0015 W, 0.0075 cm^2^).

## 5. Conclusions

Light at different wavelengths and intensities is able to improve endothelial function. The effect is correlated to the primary targets of red and NIR light within the cell, which changes leads to secondary cellular pathways activation responsible for modulation of inflammation, angiogenesis, and vasodilatation. However, PBM is a therapy derived by a complex mixture of wavelengths with different frequencies, amplitudes, and energies, which are absorbed, scattered, and reflected by biological material. Therefore, selecting the most inclusive therapy is not easy. However, 808 nm and 18 J (0.2 W, 2.05 cm^2^) intracoronary irradiation during percutaneous coronary interventions can support clinicians to prevent restenosis; as well, irradiation on two points at baseline and after 1, 3, and 7 days with 645 nm and 20 J (0.25 W, 2 cm^2^) can stimulate angiogenesis. Lastly, the use of PBM to support hypertension cure showed interesting insights that stimulate investigation by more extensive randomised controlled trials.

## Figures and Tables

**Table 1:** In vitro studies on photobiomodulation and endothelial dysfunction, selected after inclusion and exclusion criteria screening. The table shows the schematic design of the experimental setup and the results.

Cell Line	Wavelength	Parameters Irradiated	Methods	Effect of PBM
HuC EC (PCC) [29]	632 nm Laser	Power = 0.0035 W; power density = 0.0017 W/cm^2^; time = 0, 60, 180, 300, 600, 900, 1200, 1800, 2400, and 3600 s; spot area = 2 cm^2^; energy = from 0 to 12.6 J; fluence = from 0 to 6.3 J/cm^2^; mode = continuous wave (CW)	No. of irradiations = 1. Investigation setup: proliferation and human VEGF immunoassay	Stimulation of endothelial cell growth; increment of VEGF secretion by 0.63, 1.05, 2.1, and 4.2 J; no effect by 0.21 and 6.3 J; decrement of VEGF secretion by 8.4 and 12.6 J
RVM-neo (PCC) [26]	670 nm LED	Power = 0.005, 0.025, and 0.05 W; power density = 0.005, 0.025, and 0.05 W/cm^2^; time = 300 s; spot area = 1 cm^2^; energy = 1.5, 7.5, and 15 J; fluence = 1.5, 7.5, and 15 J/cm^2^; mode = CW	No. of irradiations = 1 with incremented energies (1.5, 7.5, and 15 J) in LDH and Casp experiments. 1 with fluence 7.5 J/cm^2^ in other experiments. Investigation setup: cell viability; Casp 3 assay; flow cytometer for annexin V, cytochrome c release assay; measurement of intracellular NO, oxygen consumption, ATP synthesis	Decrement of apoptosis indices (casp 3, annexin, cytochrome c); increment of ATP and NO production
RAEC (PCC) [30]	685 nm Laser	Power = 0.02 W, Power density = 0.011 W/cm^2^, Time = 720 s, Spot area = 1.8 cm^2^, Energy = 14.4 J, Fluence = 8 J/cm^2^, Mode = continuous wave (CW)	No. of irradiations = 4 (12, 24, 36, and 48 h after plating). Investigation set-up: proliferation assay; immunohistochemistry for actin filaments	Stimulation of cellular proliferation; changes in the cytoskeleton through the reorganization of actin filaments and neo-formation of stress fibres
h-AFC (PCC) [31]	645 nm Laser	Power = 0.025, 0.010, 0.012 W; power density = 0.009, 0.003, 0.004 W/cm^2^; time = 640, 1591, 1257 s to generate 6 J; time = 1278, 3183, 2515 s to generate 32 J; time = 2559, 6366, 5029 s to generate 64 J; spot area = 2.8 cm^2^; energy = 16, 32, and 64 J; fluence = 5.76, 11.51, 23.02 J/cm^2^; mode = CW	No. of irradiations = 1. Investigation set-up: cell cytotoxicity and LDH release assay. Immunofluorescence and qRT-PCR: TNF-α, IL-1β, MMP1, MMP3, IL-6, IL-8, VEGF-A, VEGF-B, VEGF-C, NGF, and BDNF	Decrement of inflammatory mediators, and catabolic enzymes; 32 J inhibited MMP1, MMP3 BDNF; 64 J inhibited MMP1, MMP3, BDNF, IL-8
HPF (PCC) [32]	660 nm Laser	Power = 0.01 W or 0.015 W; power density = 0.25 or 0.37 W/cm^2^; time = 10 s; spot area = 0.04 cm^2^; energy = 0.1 or 0.15 J; fluence = 2.5 or 3.7 J/cm^2^; mode = CW	No. of irradiations = 1. Investigation setup: viability and proliferation assay; ELISA, VEGF-C, VEGF-A, VEGFR2, FGF-2, PDGF, VEGFR1, PECAM-1, VEGF-D, PLGF, BMP-9	Both energy increased secretion of VEGF-A, VEGF-C, and VEGFR1; upregulation of BMP-9; downregulation of PDGF by both energies; 0.1 J was better than 0.15 J for capillary-like structure formation
HUVEC^(PCC)^ [33]	623.5 nm Laser	Power = 0.0013 W; power density = 0.0001 W/cm^2^; time = s; spot area = 9.06 cm^2^; energy = 0.78, 2.34, 4.68, 7.02; fluence = 0.086, 0.26, 0.52, 0.77 J/cm^2^; mode = CW	No. of irradiations = 1. Investigation setup: nitrate and nitrite Griess Assay; qRT-PCR, eNOS; Western Blot Analysis, eNOS and vinculin; migration assay; tube formation analysis	Upregulation of eNOS expression through PI3K pathway and increment of vinculin protein; cell migration promoted with 2.34 J
HUVEC [34]	660, 670, 820, 808 nm Laser	Power = 0.01, 0.02, 0.04, 0.1, 1.5 W; power density = 0.028, 0.057, 0.11, 0.28, 4.28 W/cm^2^; time = from 5 to 100 s; spot area = 0.35 cm^2^; energy = from 0.05 to 150 J; fluence from 0.14 to 428 J/cm^2^; mode = CW	No. of irradiations = 1. Investigation setup: cell proliferation assay	Stimulation of cell proliferation
HUVEC^(PCC)^ [35]	635, 830 nm Laser	635-nm: Power = 0.15 W Power density = 0.00187 W/cm^2^ Time = 1066, 2133, 4266 s Spot area = 80 cm^2^, Energy = 160, 320, 640 J, Fluence 2, 4, 8 J/cm^2^ Mode = CW 830-nm: Power = 0.3 W Power density = 0.00375 W/cm^2^ Time = 533, 1066, 2133 s Spot area = 80 cm^2^, Energy = 160, 320, 640 J, Fluence 2, 4, 8 J/cm^2^ Mode = CW	No. of irradiations = 2 radiations on the day 2 and 4 with one day-break Investigation set-up: proliferation assay; ELISA, VEGF-A, TGF-1	635 nm increased cell proliferation and decreases VEGF-A concentration; 830 nm decreased TGF-1 concentration
HUVEC^(PCC)^ [36]	635 nm Laser	Power = 0.15 W; power density = 0.00187 W/cm^2^; time = 1066, 2133, 4266 s; spot area = 80 cm^2^; energy = 160, 320, 640 J; fluence 2, 4, 8 J/cm^2^; mode = CW	No. of irradiations = 2 radiations on day 2 and 4 with one day break. Investigation setup: proliferation assay; ELISA test, VEGF-A and presence of soluble VEGF receptors (sVEGFR-1 and sVEGFR-2)	Decrement of VEGF-A, sVEGFR-1, and sVEGFR-2; activation of cell proliferation
HUVEC^(PCC)^ hyperglycemia [37]	635, 830 nm Laser	635 nm: Power = 0.15 W; power density = 0.00187 W/cm^2^; time = 1066 s; spot area = 80 cm^2^; energy = 160 J; fluence 2 J/cm^2^; mode = CW 830 nm: Power = 0.3 W; power density = 0.00375 W/cm^2^; Time = 533 s; spot area = 80 cm^2^; energy = 160 J; fluence 2 J/cm^2^; mode = CW	No. of irradiations = 2 on day 5 and 6. Investigation setup: induction of hyperglycemia; proliferation assay; ELISA, TNF-α and IL-6	Increment of proliferation; reduction of inflammation by decrease of TNF-α and IL-6; 830 nm affect more than 635 nm
HUVEC^(PCC)^ hyperglycemia [38]	635, 830 nm Laser	635 nm: Power = 0.15 W; power density = 0.00187 W/cm^2^; time = 1066 s; spot area = 80 cm^2^; energy = 160 J; fluence 2 J/cm^2^; mode = CW 830 nm: Powe r = 0.3 W; power density = 0.00375 W/cm^2^; time = 533 s; spot area = 80 cm^2^; energy = 160 J; fluence 2 J/cm^2^; mode = CW	No. of irradiations = 2 on day 5 and 6. Investigation setup: induction of hyperglycemia; proliferation assay. ELISA, sE-selectin and sVCAM	Decrement in sE-selectin and sVCAM concentration; increment of proliferation; 830 nm affect more than 635 nm
HUVEC [39]	650 nm Laser	Power = ~2 W; power density = 0.031, 0.011, 0.002 W/cm^2^; time = from 16 to 1920 s; spot area = 63.6, 191, 961.6 cm^2^; energy = from 32 to 3,840 J; fluence = from 0.5 to 20 J/cm^2^; mode = CW	No. of irradiations = 1. Investigation setup: proliferation assay; scratch test; tube formation assay; Western blot analysis, PI3K, p-PI3K, Akt, p-Akt, VEGF-A, eNOS, HIF-1α. ELISA, VEGF-A	Increment of proliferation, migration, and tube formation; activation of by PI3K/Akt signalling pathway
HUVEC [40]	660, 780 nm Laser	Power = 0.04 W; power density = 1 W/cm^2^; time = 1, 5, 10, 20 s; spot area = 0.04 cm^2^; energy = 0.04, 0.2, 0.4, 0.8 J; fluence 1, 5, 10, 20 J/cm^2^; mode = CW	No. of irradiations = 1. Investigation setup: cell viability assay; total protein measure	660 nm induced increment of viability and concentration of total proteins; 0.2 and 0.4 J had better effect; 780 nm had inhibitory effect.
L929 [41]	904 nm Laser	Repetition rate = 10 KHz; output power = 50 mW; pulse width = 100 ns; peak power = 50 W; spot area = 0.01 cm^2^; active cycle of 0.1%; energy = 200 J or 300 J; fluence = 2 J/cm^2^ or 3 J/cm^2^	No. of irradiations = 2 for two consecutive days. Investigation setup: qRT-PCR, COL1α1, VEGF	200 J incremented expression of the genes COL1α1 and VEGF; 300 J incremented expression of the genes VEGF
EA.hy926 [42]	660 nm Laser	Power = 1 W; power density = 0.003 W/cm^2^; time = 3600 s; spot area = 314 cm^2^; energy = 3600 J; fluence = 11. 5 J/cm^2^; mode = CW	No. of irradiations = 1 for proliferation. 1 + 1 irradiation after 24 h for other experiments. Investigation setup: Western blotting, phospho-ERK, ERK, phosphop38, p38, phospho-JNK, JNK, NF-κB, iNOS, and cleaved caspase 3/8/7/9, PARP	Protection against TNF/CHX-induced apoptosis by inhibition of p38 MAPK and NF-κB signals
HMVEC-d [43]	670 nm Laser	Power = 0.025 W, 0.05 W or 0.1 W; power density = 0.025, 0.05 or 0.1 W/cm^2^; time = 30, 60 or 120 s; spot area = 1 cm^2^; energy = 0.75, 1.5, 3, 6, or 12 J; fluence = 0.75, 1.5, 3, 6, 12 J/cm^2^; mode = CW	No. of irradiations = 1. Investigation setup, NO production	Increment of NO release from bound substances in healthy and diabetic model
HECV-d [44]	808 nm Laser	Power = 0.95 W; power density = 0.95 W/cm^2^; time = 60 s; spot area = 1 cm^2^; energy = 57 J; fluence = 57 J/cm^2^; mode = CW	No. of irradiations = 1 irradiation. Investigation setup: cell viability, lipid peroxidation, scratch-wound healing assay; nitrite/nitrate quantification; Western blotting, NF-kB p65; oxygen consumption measurements; ATP synthase activity assay	Increment of proliferation and migration; moderate increase in ROS productionm no increment in oxidative or nitrosative stress and NF-kB p65; shift from anaerobic to aerobic metabolism and increment of ATP

Legend: Akt, alpha serine/threonine kinases; ATP, adenosine triphosphate; BDNF, brain-derived neurotrophic factor; BMP, bone morphogenetic protein; Casp, caspase; COL, collagen type; EA.hy926, permanent human umbilical vein cell line; ELISA, enzyme-linked immunosorbent assay; eNOS, endothelial nitric oxide synthase; ERK, extracellular signal-regulated kinase; FGF, fibroblast growth factor; h-AFC, human adjacent annulus fibrosus cells; HECV, human endothelial cells; HMVEC-d, human dermal microvascular endothelial cells; HIF, hypoxia-inducible factors; HPF, human pulp fibroblasts; HuC EC, human endothelial cells isolated from coronary vessels; HUVEC, human umbilical vein endothelial cells (immortalised); HUVEC^(PCC)^, human umbilical vein endothelial cells from primary cell culture; IL, interleukin; iNOS, inducible nitric oxide synthase; JNK, c-Jun N-terminal kinase; L929, mouse NCTC clone 929 Clone of strain L; LDH, lactate dehydrogenase; MAPK, mitogen-activated protein kinase; MMP, matrix metalloproteinase; NF-κB, nuclear factor-κB; NGF, nerve growth factor; NO, nitric oxide; PARP, poly (ADP-ribose) polymerase; PCC, primary culture cells; PDGF, platelet derived growth factor; PECAM, platelet endothelial cell adhesion molecule; PI3K, phosphoinositide 3-kinases; PLGF, placental growth factor; qRT-PCR, real-time quantitative reverse transcription; RAEC, rabbit aorta endothelial cell line; ROS, reactive oxygen species; RVM-neo, neonatal rat ventricular myocytes; TGF, transforming growth factor; TNF-α, tumour necrosis factor alpha; TNF/CHX, tumour necrosis factor/cycloheximide; VEGF, vascular endothelial growth factor; VEGFR, vascular endothelial growth factor receptor; sVCAM, soluble vascular cell adhesion molecule; YAP/TAZ, yes-associated protein/transcriptional coactivator with PDZ-binding motif.

**Table 2:** Preclinical in vivo studies on photobiomodulation and endothelial dysfunction, selected after inclusion and exclusion criteria screening. The table shows the schematic design of the experimental setup on animals and the results.

Animal Model	Wavelength	Parameters Irradiated	Methods	Effect of PBM
Rabbit (ischemia, cardiac disease) [45]	660 nm LED	Power = 0.003 or 0.06 W; power density = 0.003 or 0.06 W/cm^2^; time = 180 s; spot area = 1 cm^2^, energy = 10.8 or 0.54 J; fluence = 10.8 or 0.54 J/cm^2^; mode = continuous wave (CW)	No. of irradiations and mode = 1 irradiation and 3 or 5 cycles on 1 point with probe at 25 cm from target. Investigation setup: release of NO from nitrosyl heme proteins	Increment of NO release and cardioprotective effects
Rat (ischemia, long flaps) [46]	810 nm Laser	Power = 0.1 W; power density = 0.0314 W/cm^2^; time = 360 s; spot area = 5.28 cm^2^; energy = 59.66 J; fluence = 11.30 J/cm^2^; mode = CW	No. of irradiations and mode = 1 irradiation for 4, 7, 10, or 14 days, on 1 point with the probe in contact mode. Investigation setup: immuno- and histochemical staining, VEGF, smooth muscle actin, factor VIII	Reduction of inflammation; increment of ischemic flap revascularization and flap viability; decrement of VEGF; increment of smooth muscle actin and factor VIII
Rat (ischemia, infarction) [47]	804 nm Laser	Power = 0.0157, 0.025, 0.037, 0.053 W; power density = 0.005, 0.008, 0.012, 0.017 W/cm^2^; time = 120 s; spot area = 3.14 cm^2^, energy = 1.88, 3, 4.52, 6.40 J; fluence = 0.6, 0.96, 1.44, 2.04 J/cm^2^; mode = CW	No. of irradiations and mode = 1irradiation on 1 point on infarcted heart area. Investigation setup: infarct size and angiogenesis determination; immunoblot analysis, VEGF, iNOS	Increment of angiogenesis and cardioprotection; increment of VEGF and iNOS
Rat (ischemia, coronary) [48]	635 nm Laser	Power = 0.005 W; power density = 0.006 W/cm^2^; time = 150 s; spot area = 0.8 cm^2^; energy = 0.8 J, fluence = 1 J/cm^2^; mode = CW	No. of irradiations and mode = 1 irradiation at 26 mm above the myocardium. Investigation setup: antibody array analysis for cytokines; ELISA, cytokine antibody; echocardiographic assessments	Improvement in ischemic heart disease; modulation of granulocyte-macrophage colony stimulating factor and fractalkine
Rat (ischemia, coronary) [49]	660 nm Laser	Power = 0.015 W, power density = 0.019 W/cm^2^, time = 60 s, spot area = 0.785 cm^2^, energy = 17.66 J, fluence = 22.5 J/cm^2^, mode = CW	No. of irradiations and mode = 1 irradiation at 3 cm from target on 1 point. Investigation setup: biometric data and myocardial size; qRT-PCR and Western blot analysis, interleukins, Mas, kinin B2, and plasma kallikrein; plasma nitric oxide metabolites measurement.	Decrement of myocardium inflammation and infarct size; attenuation of left ventricle dysfunction; decrement of myocardial interleukin-1 beta, interleukin-6 and Mas receptor; increment of kinin B2 and plasma kallikrein; increment of NO derivates.
Hamster (angiogenesis, mucositis) [50]	660 nm Laser	Power = 0.0328 or 0.0962 W; power density = 10.9 or 32 W/cm^2^; time = 16 or 6 s; spot area = 0.003 cm^2^; energy = 0.52 or 0.56 J; fluence = 173 or 187 J/cm^2^; mode = continuous wave (CW)	No. of irradiations and mode = 1 irradiation on 5 points at days 3, 4, 5, and 6 of the experiment. Investigation setup: immunohistochemistry, COX-2, VEGF, factor VIII	Reduction of mucositis severity. 0.52 J decreased COX-2 and 0.56 J decreased VEGF
Mice HRS/J (angiogenesis, muscle) [51]	780 nm Laser	Power = 0.04 W; power density = 1 W/cm^2^; time = 20 s; spot area = 0.04 cm^2^; energy = 0.8 J; fluence = 20 J/cm^2^; mode = continuous wave (CW)	No. of irradiations and mode = 1 irradiation on 1 point, for 3, 6, or 10 times, on alternate days. Contact mode. Investigation setup: immunoblot, MMP; immunoistochemistry, VEGF, VEGFR-2.	No effect on MMP; decrement of VEGF and VEGFR-2; the effects are visible only after the 10th irradiation
Rat (angiogenesis, muscle) [52]	660 nm Laser	Power = 0.02 or 0.04 W; power density = 0.05 or 0.1 W/cm^2^; time = 20 s or 50; spot area = 0.4 cm^2^; energy = 0.4 or 2 J; fluence = 1 or 5 J/cm^2^; mode = CW	No. of irradiations and mode = 1 irradiation on 1 point. Treatments started 48 h post-surgery and were performed five times/week (each 24 h). Contact mode. Investigation setup: histopathological analysis; qRT-PCR, VEGF, COX-2, MyoD, myogenin	Improvement of muscle regeneration; decrement of COX-2; increment of VEGF, MyoD. Myogenin increased with 2 J
Rat (angiogenesis, muscle) [53]	904 nm Laser	Repetition rate = 9.500 Hz; output powe r = 0.04 W; pulse width = 60 ns; peak power = 50 W; spot area = 0.1 cm^2^; energy = 0.3 J or 0.5 J; fluence = 3 J/cm^2^ or 5 J/cm^2^	No. of irradiations and mode = 1 irradiation on 5 points, 2, 12, and 24 h after trauma. Probe at 0.5 cm from target. Investigation setup: Griess nitrite, lipid peroxidation, protein carbonylation, glutathione peroxidase, and catalase activity assay; dityrosine autofluorescence determination; qRT-PCR, VEGF, BDNF, IL-6, IL-10	Accelerated recovery; decrement of inflammation; decrement of IL-6; increment of IL-10; 0.3 J prevents thiobarbituric acid-reactive substance, carbonyl, superoxide dismutase, glutathione peroxidase, and catalase increment; BDNF and VEGF are prevented by irradiation
Rat (angiogenesis, skin) [54]	660 nm Laser	Power = 0.03 W; power density = 1.07 W/cm^2^; time = 67 s; spot area = 0.028 cm^2^; energy = 2 J; fluence = 72 J/cm^2^; mode = CW	No. of irradiations and mode = 1 irradiation on 1 point. Alternate days for 14 days. Contact mode. Investigation setup: immunohistochemistry, VEGF, TIMP-2, MMP-3 and -9, collagen I, and III; qRT-PCR: IL-6; ELISA, CINC-1	Accelerated recovery in early stages of tissue repair; modulation of IL-6, CINC-1, VEGF, MMP-3, MMP-9 and TIMP-2; increment of collagen
Rat (angiogenesis, skin) [55]	670 nm Laser	Power = 0.03 W; power density = 0.476 W/cm^2^; time = 30 s; spot area = 0.063 cm^2^; energy = 0.9 J; fluence = 14.28 J/cm^2^; mode = CW	No. of irradiations and mode = 1 irradiatio on 1 point. 15 consecutive days of treatment. Contact mode. Investigation setup: histological analysis; immunohistochemistry, collagen I, TNF-α, VEGF	Accelerated recovery of the cutaneous wound healing; decrement of inflammatory infiltrate and TNF-α; increment of VEGF and collagen type 1.
Rat (angiogenesis - skin) [56]	904 nm Laser	Repetition rate = 100 Hz; output power = 0.00078 W; pulse width = 200 ns; spot area = 1.77 cm^2^; time = 600 s; energy = 0.4 J; fluence = 0.2 J/cm^2^	No. of irradiations and mode = 1 irradiation on 1 point. Daily for seven days post-burn injury. Contact mode. Investigation setup: assays for oxidative stress and antioxidants markers, ROS, NO, lipid peroxidation, GSH, SOD, catalase, GPx, advanced oxidation protein products; immunoblot, Nrf2, HO-1, Txnrd2	Accelerated recovery of the burn wound healing; decrement of ROS, NO, lipid peroxidation, protein carbonylation, advanced oxidation protein product levels, GSH, and thiol (T-SH, Np-SH, P-SH); increment of endogenous antioxidants levels of SOD, catalase, GPx
Rat (angiogenesis, skin) [57]	660, 780 nm Laser	780 nm: Power = 0.07 W; power density = 1.75 W/cm^2^; time = 20 s; spot area = 0.04 cm^2^; energy = 1.4 J; fluence = 35 J/cm^2^; mode = CW 606 nm: Power = 0.04 W; power density = 1 W/cm^2^; time = 20 s; spot are a = 0.04 cm^2^; energy = 0.8 J; fluence = 20 J/cm^2^; mode = CW	No. of irradiations and mode = 1 irradiation on 2 points for two days. Probe at 1 mm from target. Investigation setup: qRT-PCR, VEGF	Accelerated recovery of wound healing; modulation of expression of VEGF
Rat (angiogenesis, skin) [58]	660 nm Laser 635 nm LED	Power = 0.04 W; power density = 0.32 W/cm^2^; time = 62 s; spot area = 0.125 cm^2^; energy = 2.5 J; fluence = 19.74 J/cm^2^; mode = CW	No. of irradiations and mode = 1 irradiation on 4 points for 2, 4, or 6 days. Contact mode. Investigation setup: histology, collagen; immunohistology, TGF-β	Stimulation of angiogenesis; increment of collagen expression; increment of blood vessels formation; TGF-β no stimulated
Rats (angiogenesis, skin) [59]	660 nm Laser	Laser: Power = 0.04 W; power density = 1 W/cm^2^; time = 31 or 126 s; spot area = 0.04 cm^2^; energy = 0.2 or 0.8 J; fluence = 5 or 20 J/cm^2^; mode = CW LED: Power = 0.09 W; power density = 1.06 W/cm^2^; time = 17 or 56 s; spot area = 0.085 cm^2^; energy = 0.42 or 1.7 J; fluence = 5 or 20 J/cm^2^; mode = CW	No. of irradiations and mode = 1 irradiation on 7 points for 2, 6, 13, or 20 days. Contact mode. Investigation setup: histology	Improvement of angiogenesis; light coherence was shown not to be essential to angiogenesis
Rats (angiogenesis, skin) [60]	660 nm Laser	Power = 0.04 W; power density = 1 W/cm^2^; time = 4 or 20 s; spot area = 0.04 cm^2^; energy = 0.16 or 0.8 J; fluence = 4 or 20 J/cm^2^; mode = CW	No. of irradiations and mode = 1 irradiation on 2 points for 14 days. Contact mode. Investigation setup: ELISA, IL-1ß and TNF-α; image analysis for micro-vessel density	Improvement of oral wound repair and angiogenesis; increment of IL-1ß and TNF-α
Rats (angiogenesis, skin) [61]	660, 780 nm Lasers	Power = 0.04 W; power density = 0.32 W/cm^2^; time = 30 or 40 s; spot area = 0.125 cm^2^; energy = 1.2 or 1.6 J; fluence = 9.6 or 12.8 J/cm^2^; mode = CW	No. of irradiations and mode = 1 irradiation on 24 points for 4 days. Contact mode. Investigation setup: image analysis for micro-vessel density; immunoblotting, HIF-1α; qRT-PCR, VEGF, gelatin zymography, MMP-2 activity	Increment of new vessels formation; Increment of HIF-1α and VEGF; decrement of MMP-2
Rats (angiogenesis, skin) [62]	670 nm Laser	Power = 0.009 W; power density = 0.031 W/^cm2^; time = 31 s; spot area = 0.28 cm^2^; energy = 0.28 J; fluence = 1 J/cm^2^; mode = CW	No. of irradiations and mode = 1 irradiation on 4 points for 4 days. Contact mode. Investigation setup: histomorphometry; immunohistochemistry, VEGF and CD31.	Improvement of late course of healing; increment of collagens and blood vessel; no effect on VEGF
Rats (angiogenesis, skin) [63]	670 nm Laser	Power = 0.009 W; power density = 0.031 W/cm^2^; time = 31 s; spot area = 0.28 cm^2^; energy = 0.28 J; fluence = 1 J/cm^2^; mode = CW	No. of irradiations and mode = 1 irradiation on 4 points for 4 days. Contact mode. Investigation setup: histomorphometry; immunohistochemistry: CD31, NG2, smooth muscle alpha actin, CD8, CD68, Ptch, Gli-2, and Ihh.	Stimulation of later stages of wound healing and angiogenesis; decrement of CD68; increment of CD8
Rats (angiogenesis, tendon rupture) [64]	660 nm Laser	Power = 0.01 or 0.04 W; power density = 0.25 or 1 W/cm^2^; time = 10 s; spot area = 0.04 cm^2^; energy = 0.1 or 0.4 J; fluence = 2.5 or 10 J/cm^2^; mode = CW	No. of irradiations and mode = 1 irradiation on 1 point for 3, 5 and 7 days. Contact mode. Investigation setup: India ink injection	Promotion of neovascularization
Aged rats (angiogenesis) [65]	830 nm Laser	Power = 0.05 W; power density = 1.8 W/cm^2^; time = 60 s; spot area = 0.028 cm^2^; energy = 3 J; fluence = 107 J/cm^2^; mode = CW	No. of irradiations and mode = 1 irradiation on 1 point, daily for seven days post injury. Contact mode. Investigation setup: Immunohistochemistry: VEGF, MMP-3, and MMP-9; histochemistry, collagen type I and III	Increment of collagen type I and III, production; downregulation of MMP-3 and MMP-9 expression; upregulation of VEGF
SHR rats (blood pressure) [66]	660 nm Laser	Power = 0.1 W; power density = 1.71 W/cm^2^; time = 56 s; spot area = 0.058 cm^2^; energy = 5.6 J; fluence = 96 J/cm^2^; mode = CW	No. of irradiations and mode = 1 irradiation on 6 different points. Transcutaneously, with skin contact at 90° angle. Investigation setup: systolic arterial pressure; NO levels evaluation	Reduction of systolic arterial pressure; increment of nitric oxide levels; no changement in heart rate
2K rats (blood pressure) [67]	660-nm Laser	Power = 0.1 W, power density = 3.57 W/cm^2^, time = 56 s, spot area = 0.028 cm^2^, energy = 5.6 J, fluence = 200 J/cm^2^, mode = CW	(abdominal region) No. of irradiations and mode = 1 irradiation on 6 points. Transcutaneously, with skin contact at 90° angle. Investigation setup: systolic arterial, diastolic arterial, mean arterial pressure, and heart rate were measured; NO levels evaluation	Induction of long lasting hypotensive effect; vasodilation by a NO dependent mechanism
2K-1C rats (blood pressure) [68]	660 nm Laser	Power = 0.1 W; power density = 0.14 W/cm^2^; time = from 1 to 186 s; spot area = 0.722 cm^2^; energy = 0.1, 0.3, 0.6, 1.2, 2.3, 4.7, 9.3, and 18.6 J; fluence = from 0.14 to 25.76 J/cm^2^; mode = CW	No. of irradiations and mode = 1 irradiation on 6 simultaneous points. Transcutaneously, with skin contact at 90° angle. Investigation setup: systolic arterial, diastolic, arterial pressure and heart rate were measured	7.2–55.8 J is the effective therapeutic window to reduce pressure and heart rate and induce a long-lasting hypotensive effect

Legend: BDNF, brain-derived neurotrophic factor; CD, cluster of differentiation; COX, cyclooxygenase; CINC, cytokine-induced neutrophil chemoattractan; ELISA, enzyme-linked immunosorbent assay; GSH, glutathione; GSx, glutathione peroxidase; Gli-2, zinc finger protein GLI2; HIF, hypoxia-inducible factors; HO-1, heme oxygenase; IL, interleukin; Ihh, Indian hedgehog homolog; iNOS, inducible nitric oxide synthase; Mas = MAS-G-protein coupled receptor; MMP = matrix metalloproteinase; MyoD = myoblast determination protein; Nrf2 = nuclear factor erythroid 2-related factor; NO = nitric oxide Ptch = protein patched homolog; qRT-PCR = Real-Time quantitative reverse transcription; ROS = reactive oxygen species; SOD = superoxide dismutase; TGF = transforming growth factor; TIMP = tissue inhibitors of metalloproteinases; TNF-α = tumour necrosis factor alpha; Txnrd2 = thioredoxin reductase; VEGF = vascular endothelial growth factor; VEGFR = vascular endothelial growth factor receptor.

**Table 3:** Clinical in vivo studies on photobiomodulation and endothelial dysfunction, selected after inclusion and exclusion criteria screening. The table shows the schematic design of the experimental set-up on patients and the results.

Study/Disease	Wavelength	Parameters Irradiated	Methods	Effect of PBM
91 Patiens (RCT) (angioplasty) [69]	808 nm Laser	Power = 0.2 W, power density = 0.1 W/cm^2^, time = 90 s, average spot area = 2.05 cm^2^, energy = 18 J, fluence = ~9 J/cm^2^, mode = continuous wave (CW)	No. of irradiations and mode = 1 intracoronary irradiation during percutaneous coronary interventions. Investigation setup: serum levels of IGF-1, VEGF, TGF and FGF-2 were measured before angioplasty, then, 6 and 12 h and 1 month after the procedure	Smaller neointima formation; IGF-1 and VEGF = no-effect; decrement fo FGF-2; increment of TGF-b1
101 Patients (RP) (angioplasty) [70]	808 nm Laser	Power = 0.2 W, power density = 0.1 W/cm^2^, time = 90 s, average spot area = 2.05 cm^2^, energy = 18 J, fluence = ~9 J/cm^2^, mode = CW	No. of irradiations and mode = 1 intracoronary irradiation during percutaneous coronary interventions. Investigation setup: serum levels of NO derivates and endothelin-1 were measured before angioplasty, then, 6 and 12 h and 1 month after the procedure	Improvement of restenosis process; increment of NO derivates; endothelin-1 increased after 6 h but decreased later
14 Patients (PS) (saphenectomy) [71]	780 nm laser	Power = 0.025 W, power density = 0.625 W/cm^2^, time = 30 s, average spot area = 0.04 cm^2^, energy = 0.75 J, fluence = 19 J/cm^2^, mode = CW	No. of irradiations and mode = 1 irradiation surrounding the entire surgical perimeter wound edge. Investigation setup: evaluation of erythema, edema, blister, hematoma, transudation, dehiscence, and pain	Prevention of prodromal complications in saphenectomy post myocardial revascularization
27 Patients (PS) (cerebral ischemia) [75]	633 nm laser	Power = 0.025 or 0.045 W, power density = 0.14 or 0.045 W/cm^2^, time = 1200 or 2400 s, average spot area = 0.18 cm^2^, energy = 29, 54, 60 or 106 J, fluence = from 161 to 589 J/cm^2^, mode = CW	No. of irradiations and mode = 1 intracerebral transcatheter laser irradiation. Investigation setup: restoration of mental and motor functions was detected; rheoencephalography, scintigraphy, computed tomography and magnetic resonance imaging was performed	Restoration of cerebral collateral and capillary blood supply; improvement of microcirculation; restoration of cellular and tissue metabolism; stimulation of neurogenesis and regenerative processes
21 aged Patients (PS) (cerebral ischemia) [73]	1064 nm Laser	Power = 3.4 W, power density = 0.25 W/cm^2^, time = 240 s, spot area = 13.6 cm^2^, energy = 816 J, fluence = 60 J/cm^2^, mode = CW	No. of irradiations and mode = 1 irradiation at the right forehead on 2 points. Investigation setup: prefrontal cortex measures of attention PVT and memory, carotid artery intima-media thickness, electroencephalography, and functional magnetic resonance imaging	Improvement of cognitive performance and both carotid artery and intima-media thickness; increment and improvement of resting-state EEG alpha, beta, and gamma power as well as prefrontal bloodoxygen- evel
7 diabetic Patients (PS) (angiogenesis/healing) [72]	660 nm Laser	Power = 0.1 W, power density = 0.16 W/cm^2^, time = 12 s, spot area = 0.6 cm^2^, energy = 1.2 J, fluence = 2 J/cm^2^, mode = CW	No. of irradiations and mode = 1 irradiation around lesion area; 0.5 cm distant from tissue; points were 2 cm far from each other. Investigation setup: qRT-PCR, IL6, TNF, VEGF, and TGF	IL6 not changed; decrement of TNF; increment of VEFG and TGF-ß
40 Patients (PS) (angiogenesis/healing) [73]	645 nm Laser	Power = 0.25 W, power density = 0.125 W/cm^2^, time = 80 s, spot area = 2 cm^2^, energy = 20 J, fluence = 10 J/cm^2^, mode = CW	No. of irradiations and mode = 1 irradiation on 2 points at baseline and after 1, 3, and 7 days. Investigation setup: ELISA on crevicular fluid, bradykinin, VEGF and EGF	Improvement of the early phases of the healing and agiogenesis; reduction of bradykinin and VEGF; increment of EGF.
10 Patients (PS) (angiogenesis/healing) [74]	808 nm Laser	Power = 0.05 W, power density = 1.6 W/cm^2^, time = 400 s, spot area = 0.031 cm^2^, energy = 20 J, fluence = 645 J/cm^2^, mode = CW	No. of irradiations and mode = 1 irradiation once a day for three consecutive days. Investigation setup: blood analysis for VEGF, FGF, angiostatin, GSH, symmetric dimethyl-arginine, asymmetric dimethylarginine and L-arginine	No change in VEGF, FGF, SDMA, NO, and ADMA levels; increment of antioxidant and angiogenic potential.
30 Patients (PS) (hypertension) [76]	635 nm Laser	Power = 0.0015 W, Power density = 0.2 W/cm^2^, Time = 900 s, spot area = 0.0075 cm^2^, Energy = 1.35 J, Fluence = 180 J/cm^2^, Mode = CW	No. of irradiations and mode = 1 intravein irradiation for 10 procedures. Investigation setup: endothelium function was evaluated by test with reactive hyperemia	Decrement of cardiac risk; decrement of DayDBP in hyperuricemia group and both DaySBP and DayDBP in the group of patients with AH combined with hyperuricemia

Legend: ADMA, asymmetric dimethylarginine; AH, arterial hypertension; DBP, diastolic blood pressure EEG, electroencephalogram; EGF, epidermal growth factor; ELISA, enzyme-linked immunosorbent assay; FGF, fibroblast growth factors; GSH, glutathione; IGF, insulin-like growth factor; IL, interleukin; NO, nitric oxide; PVT, psychomotor vigilance task; PS, pilot study; qRT-PCR, real-time quantitative reverse transcription; RCT, randomised controlled trial; RT, randomised trial; SBP, systolic blood pressure; SDMA, symmetric dimethylarginine; TGF, transforming growth factor; TNF, tumour necrosis factor; VEGF, vascular endothelial growth factor.

## Data Availability

Data available on request from the authors.

## Supplementary Materials

The following are available online at https://www.mdpi.com/2227-9059/9/3/274/s1, Figure S1. Flow chart demonstrating the selection process.
