# Supplementary figures and images for: Experimental and Clinical Applications of Red and Near-Infrared Photobiomodulation on Endothelial Dysfunction: A Review
Source: Biomedicines. 2021 Mar 9;9(3):274. doi: 10.3390/biomedicines9030274 (PMC7998572; doi:10.3390/biomedicines9030274)

# Supplementary Materials

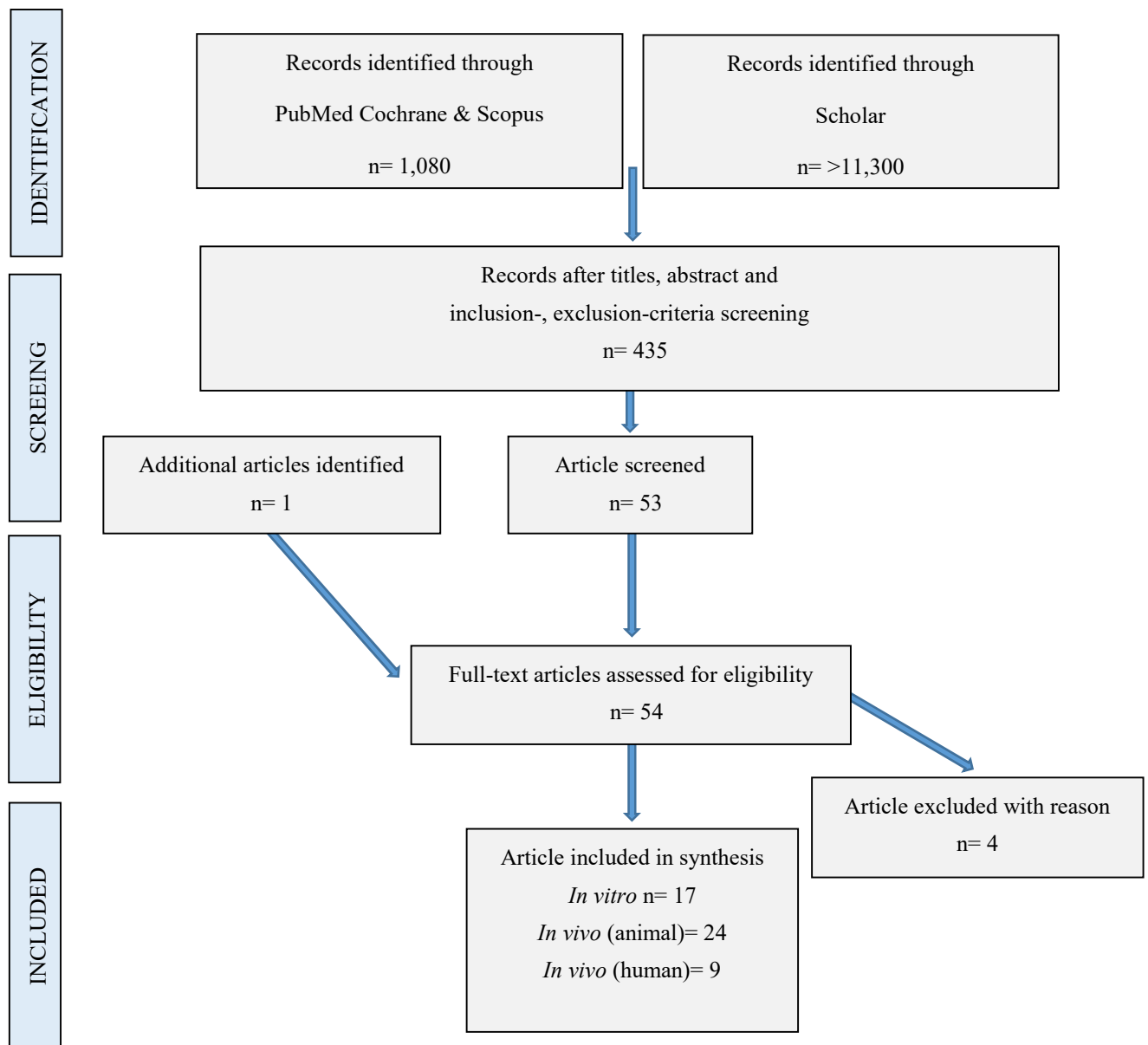

Figure S1: Flow chart demonstrating the selection process.

Supplement: Supplementary file 1 [file biomedicines-09-00274-s001.pdf]
